# Descriptive Analysis of Mobile Apps for Management of COVID-19 in Spain and Development of an Innovate App in that field

**DOI:** 10.1038/s41598-022-22601-6

**Published:** 2022-10-25

**Authors:** Isabel Herrera Montano, Javier Pérez Pacho, Santos Gracia Villar, Silvia Aparicio Obregón, Jose Manuel Breñosa Martinez, Isabel de la Torre Díez

**Affiliations:** 1 Department of Signal Theory and Communications, and Telematics Engineering, University of Valladolid, Paseo de Belén, 15, 47011 Valladolid, Spain; 2 Universidad Europea del Atlántico, Isabel Torres 21, 39011 Santander, Spain; 3 Universidad Internacional Iberoamericana, Campeche, Mexico; 4 Universidade Internacional do Cuanza Bairro Kaluanda, EN 250, Cuito, Bié Angola

**Keywords:** Information technology, Software, Diseases, Health care, Epidemiology

## Abstract

To address the current pandemic, multiple studies have focused on the development of new mHealth apps to help in curbing the number of infections, these applications aim to accelerate the identification and self-isolation of people exposed to SARS-CoV-2, the coronavirus known to cause COVID-19, by being in close contact with infected individuals. The main objectives of this paper are: (1) Analyze the current status of COVID-19 apps available on the main virtual stores: Google Play Store and App Store for Spain, and (2) Propose a novel mobile application that allows interaction and doctor-patient follow-up without the need for real-time consultations (face-to-face or telephone). In this research, a search for eHealth and telemedicine apps related to Covid-19 was performed in the main online stores: Google Play Store and App Store, until May 2021. Keywords were entered into the search engines of the online stores and relevant apps were selected for study using a PRISMA methodology. For the design and implementation of the proposed app named COVINFO, the main weaknesses of the apps studied were taken into account in order to propose a novel and useful app for healthcare systems. The search yielded a total of 50 apps, of which 24 were relevant to this study, of which 23 are free and 54% are available for Android and iOS operating systems (OS). The proposed app has been developed for mobile devices with Android OS being compatible with Android 4.4 and higher. This app enables doctor-patient interaction and constant monitoring of the patient's progress without the need for calls, chats or face-to-face consultation in real time. This work addresses design and development of an application for the transmission of the user's symptoms to his regular doctor, based on the fact that only 16.6% of existing applications have this functionality. The COVINFO app offers a novel service: asynchronous doctor-patient communication, as well as constant monitoring of the patient’s condition and evolution. This app makes it possible to better manage the time of healthcare personnel and avoid overcrowding in hospitals, with the aim of preventing the collapse of healthcare systems and the spread of the coronavirus.

## Introduction

In March 2020, the WHO (World Health Organization) declared the covid as pandemic^1,2^. This disease has caused economic, social, and health imbalances in all countries of the world. Governments have been forced to take strict measures to control the spread of the virus and minimize morbidity and mortality. Consequently, in an attempt to prevent the collapse of healthcare systems, citizens have been advised or forced to stay at home and practice social distancing as a primary measure to prevent the spread of COVID-19^3–5^.

The development of the Internet and mobile devices has brought about changes of great magnitude and benefit in everyday life: the immediacy of acquiring and receiving information, the increase and improvement of communication between people, the renewal of commerce, the worldwide availability of a great deal of knowledge and the equality of cultural and educational acquisition^6–8^. In the healthcare field, this evolution has had no less influence, since, mobile devices (smartphones and tablets) have been widely adopted by medical professionals, progressively becoming one of the best options for accessing clinical information, as well as, the management of various chronic diseases through mobile applications^9–11^.

The mHealth is precisely that, the branch of eHealth aimed at the development of medical practice in mobile devices that allow monitoring, prevention, diagnosis and support the treatment of different pathologies, in addition to allowing more immediate and effective doctor-patient communication. Therefore, to face the current pandemic, this advance in technology and telemedicine has been an important source of support, given the need for application-based solutions to reduce the risk of transmission by close contact and the importance of remote monitoring (through a mobile app) of the evolution of patients with Covid-19, to avoid new infections and the collapse of health units^12–17^. Multiple studies have focused on the realization of new mHealth apps to help curb the number of contagions, these apps aim to accelerate the identification and self-isolation of people exposed to SARS-CoV- 2, the coronavirus known to cause COVID-19, by being in close contact with infected individuals^7,9,18–20^.

**Figure 1:**
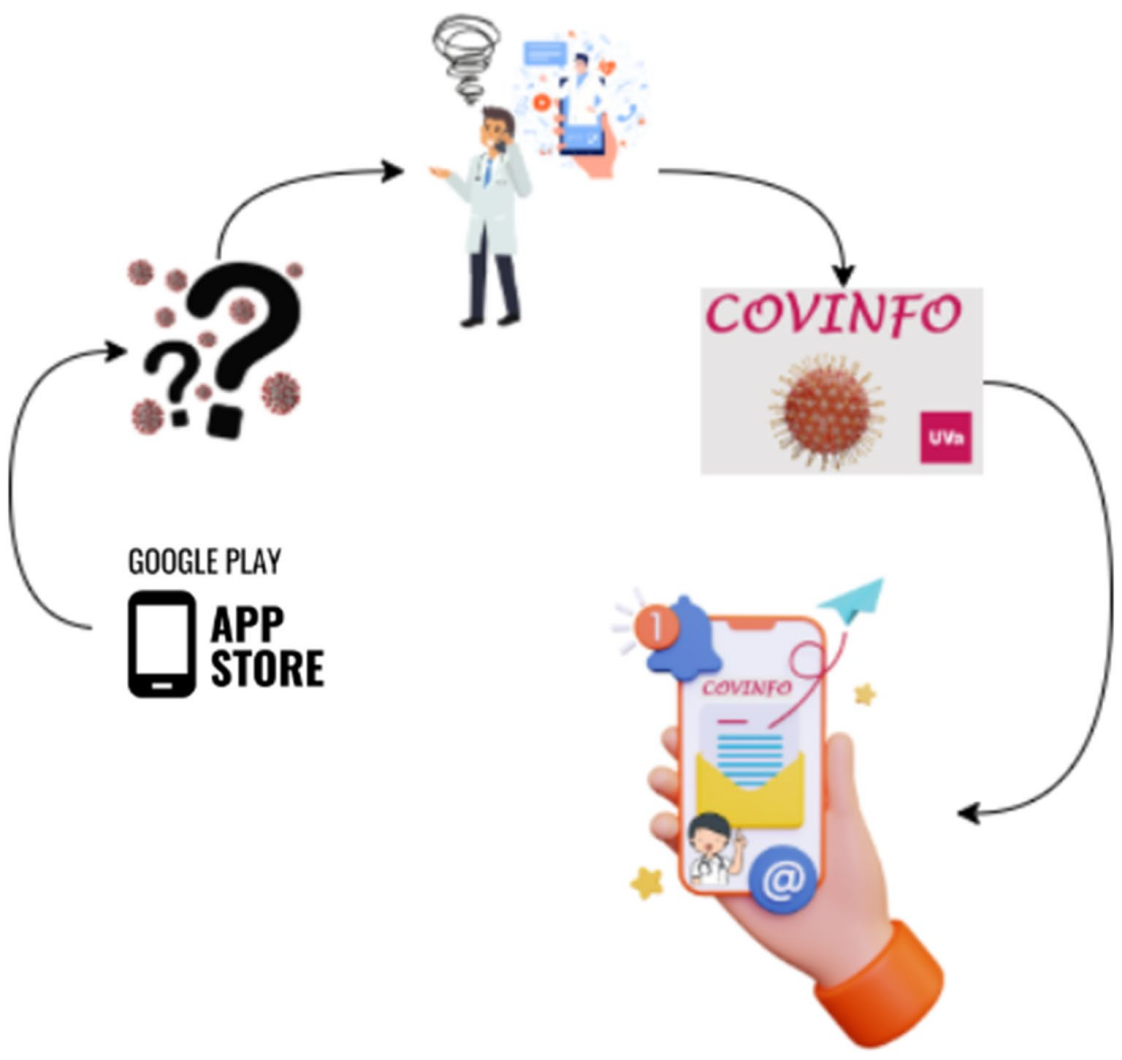
Graphical representation of the proposed objectives.

With the rapid evolution of the virus and the need to provide timely solutions, most of the apps have been created without a previous study of the existing ones^21,22^, therefore, the main objectives of this paper are: (1) Analyze the current status of COVID-19 apps available in the main virtual stores: Google Play Store and App Store for Spain, and (2) To develop a novel mobile app that allows interaction and doctor-patient follow-up without the need for real-time consultations (face-to-face or telephone). Figure 1 shows the graphic representation of the proposed objectives, where three clearly identified stages are presented: the search and analysis of the existing apps in the mobile app stores, the problem identified in them and the proposed solution.

### Related works

Related papers found base their studies on analyzing most of the COVID-19 contact tracing applications^23^, first published in August 2020; shed light on the studies found in the scientific literature that have used and evaluated mobile applications for the prevention, management, treatment or monitoring of COVID-19^24^, first published in August 2020; identify smartphone applications designed to address the COVID-19 pandemic and analyze its characteristics^22^, first released in May 2020; describe the unique benefits and challenges of adapting an internally developed application to facilitate remote communication and rounding during COVID-19^18^; analyze the evidence base on applications that were developed in response to COVID-19^25^, first published in May 2020; identify, analyze and categorize COVID-19 related health apps that are currently available to consumers in app stores; in particular, focuses on exploring their key technical characteristics and classifying the purposes for which these apps were designed^21^, first published in September 2020.

As we have seen, to date no study has been found that proposes a novel application based on the analysis of existing Covid-19 related apps in the application stores for mobile devices to ensure a new approach and usability of mHealth. Therefore, the main contribution of this research is the analysis of Covid-19 related apps and the proposal of a novel app whose support improves mHealth functionality and doctor-patient interaction, trying to place it in the stratum of consideration as a healthcare product.

The rest of the paper is structured as follows: Next, the methodology followed for the realization of this research is described. Subsequently, a section is dedicated to the results obtained in the search for apps and the proposed app is presented. Then, the results are discussed, as well as the findings and limitations of the proposed app with respect to the apps studied. Finally, the conclusions obtained in this research are presented and future lines of work are proposed.

## Methods

This study focuses on the search and selection of mobile apps related to the Covid-19 epidemic in order to develop a novel app that allows doctor-patient communication and monitoring of their evolution. This section describes the methodology followed for the search and selection of the analyzed apps, as well as for the development of the proposed new app.

### Searching and selection

**Figure 2:**
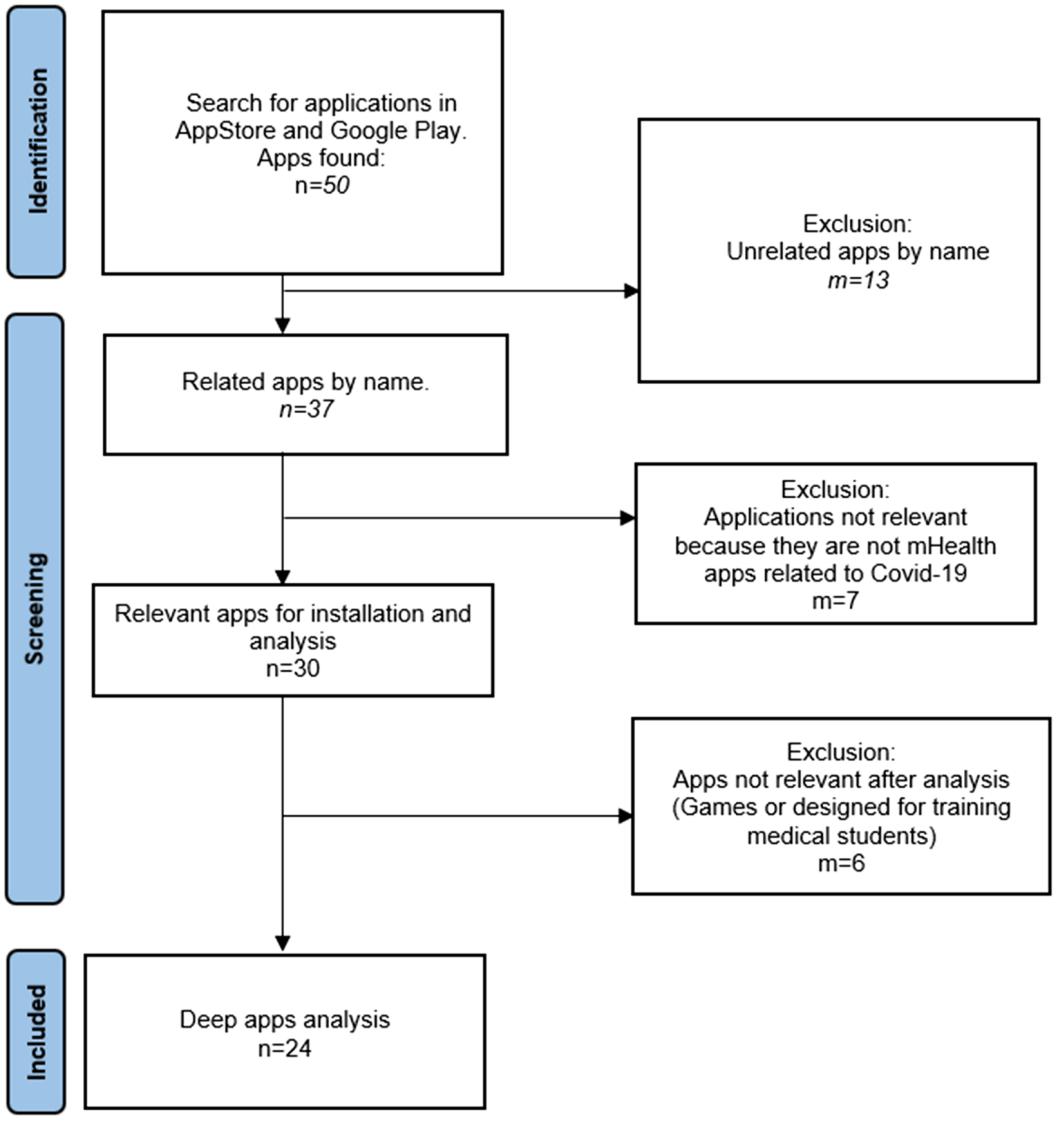
PRISMA methodology for apps selection.

The search and selection of apps was performed following a PRISMA^26^ methodology as shown in Fig. 2. The search was performed in the leading app stores in the mobile device market: App Store and Google Play Store, as in the studies^21^ and^22^, but in the period from January 2019 to May 2021, using the keywords: 'Coronavirus', 'COVID', 'COVID-19', 'SARS-COV-2'. It is important to note that these app stores limit the search according to the availability of the apps for the country from which the search is made, in this case Spain^27,28^. The keywords were entered into the App Store and Google Play Store search engines, obtaining a total of 50 apps. Then apps related to Covid-19 were selected according to name and category (the latter related to health and medicine). After applying those selection criteria in reading the description of each app, 30 are selected for installation and analysis. A further 6 apps were discarded because they were games or apps designed for training medical students, leaving a total of 24 apps for in-depth analysis.

The inclusion and exclusion criteria took into account criteria related to the objective and purpose of the apps found, for example, apps for monitoring and follow-up of the evolution of those infected, security measures, information, communication with physicians, etc. In addition, only apps developed in Spanish or English were included, although some of them have additional languages. No restrictions were established as to country of origin, price, source, developer or type of consumers.

For the analysis of the apps, they were downloaded to mobile devices with iOS version 12 and Android version 11 OS. The apps were classified according to their purpose in the categories of Information, Location/tracking, Self-assessment, Communication with physicians, and Outbreak tracking in the healthcare environment, analyzing their functioning. They were assessed according to the criteria of language, OS, keyword search result, number of downloads and consumer assessment.

The main limitations of the apps found were determined based on consumer judgment (gathered from app reviews) and our experience with handling them. This led to the development of the main features of our proposal.

### Materials and methods applied in the development of the app

The main objective of the proposed app is the multiple transmission of information about the patient's symptoms from the application, which in turn includes the history of its evolution, so that, from the screen where the physician receives the data, he can follow the patient's evolution.

For the development of the app, the Android Studio^29^ programming platform was chosen for its implementation on the Android OS since it is the most used globally in recent years and everything points to its continuity as a general rule^30^. In addition, a relational database was created with SQLite^31^ to store the information collected by the app. This information is stored in clear in the internal memory of the mobile device. Encryption algorithms will be implemented in future versions of this app to ensure that the information is stored and sent encrypted.

For all texts entered, a file called Strings.xml has been used, which will allow the application to be translated into any language quickly and easily in the future, simply by duplicating this file and translating it.

### Ethical approval

This article does not contain any studies with human participants or animals performed by any of the authors.

## Results

In this session, the results obtained in the selection of the relevant apps for this study are presented and the new app named COVINFO.

### mHealth apps focused on COVID-19

After applying the methodology described above, 24 apps were obtained as a result. Table 1 shows a summary of the apps found, developers, the OS, number of downloads, and user ratings in the app store.

**Table 1:** Summary of the apps found.

App name	Developer	OS	Downloads	Rate
COVID-19.eus^32^	Osakidetza	iOS Android	50,000	3
GVA Responde^33^	Generalitat Valenciana	iOS Android	10,000	4.25
Asistencia COVID-19^34^	Ministerio de Asuntos Económicos y transformación digital	iOS Android	50,000	3.2
CoronaMadrid^35^	Comunidad de Madrid	iOS Android	50,000	3.15
CoronaTestNavarra^36^	Gobierno de Navarra	iOS Android	5000	3.9
GVA Coronavirus^37^	Generalitat Valenciana	iOS Android	10,000	2.85
STOP COVID19 CAT^38^	Generalitat de Catalunya	iOS Android	500,000	3.7
CONFINAPP^39^	Generalitat de Catalunya	iOS Android	10,000	3.15
OpenWHO: Knowledge for health emergencies^40^	HPI Knowledge Engineering Team	Android	1,000,000	4.3
WHO Info^41^	World Health Organization	iOS Android	100,000	3.7
Radar COVID^42^	Ministerio de Asuntos Económicos y transformación digital	iOS Android	1,000,000	3.55
Covclear^43^	GMV Sistemas	iOS Android	–	–
PassCOVID.gal^44^	Xunta de Galicia	iOS Android	5000	2.8
Ser + contra COVID19^45^	AppAndAbout	iOS	1000	5
Salud Andalucia^46,47^	Sistema sanitario público de Andalucía	iOS Android	10,000	2.9
AyudaCovid^48^	Ing. Investigación e innovación para internet	iOS	100	–
HEpiTracker^49^	Fundacion Teofilo Hernando	iOS	100	–
Interactive Clinics^50^	Bit Genoma Digital Solutions SL	iOS	100	5
Mediknor^51,52^	Teckel Medical SL	iOS	300	4.7
COVID-19!^53^	Nemocnice	iOS	100	2.3
Coronavirus-Covid-19^54^	Verbaclinic	iOS	100	4.4
TousAntiCovid^55^	Gouvernement Fracais	iOS	200	2.5
Coronavirus-Extrem^56^	Gobierno de Extremadura	Android	100	3.1
Salud Covid^57^	Ing. Investigación e innovación para internet	iOS	100	2.4

Table 1 shows that a large number of the apps found have been created by the different Spanish autonomous communities and organizations, which demonstrates Spain's interest in this type of app. In addition, it is important to note that only one of the apps found is paid, Covclear^58^, the others are free.

**Figure 3:**
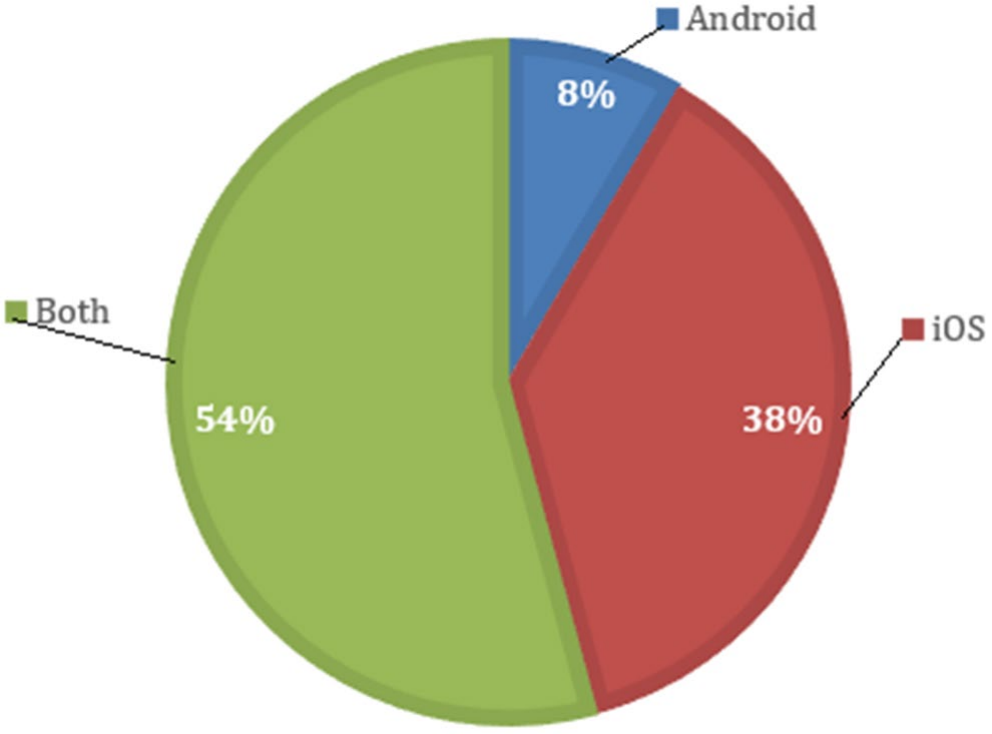
Percentage of availability of apps by OS.

As shown in Fig. 3, it can be seen that 8% of the apps found are only available for the Android OS, 38% only for iOS, and the remaining 54% are available for both OS. This means that in more than 50% of the apps, the OS of the user's mobile device is not an impediment to access.

**Figure 4:**
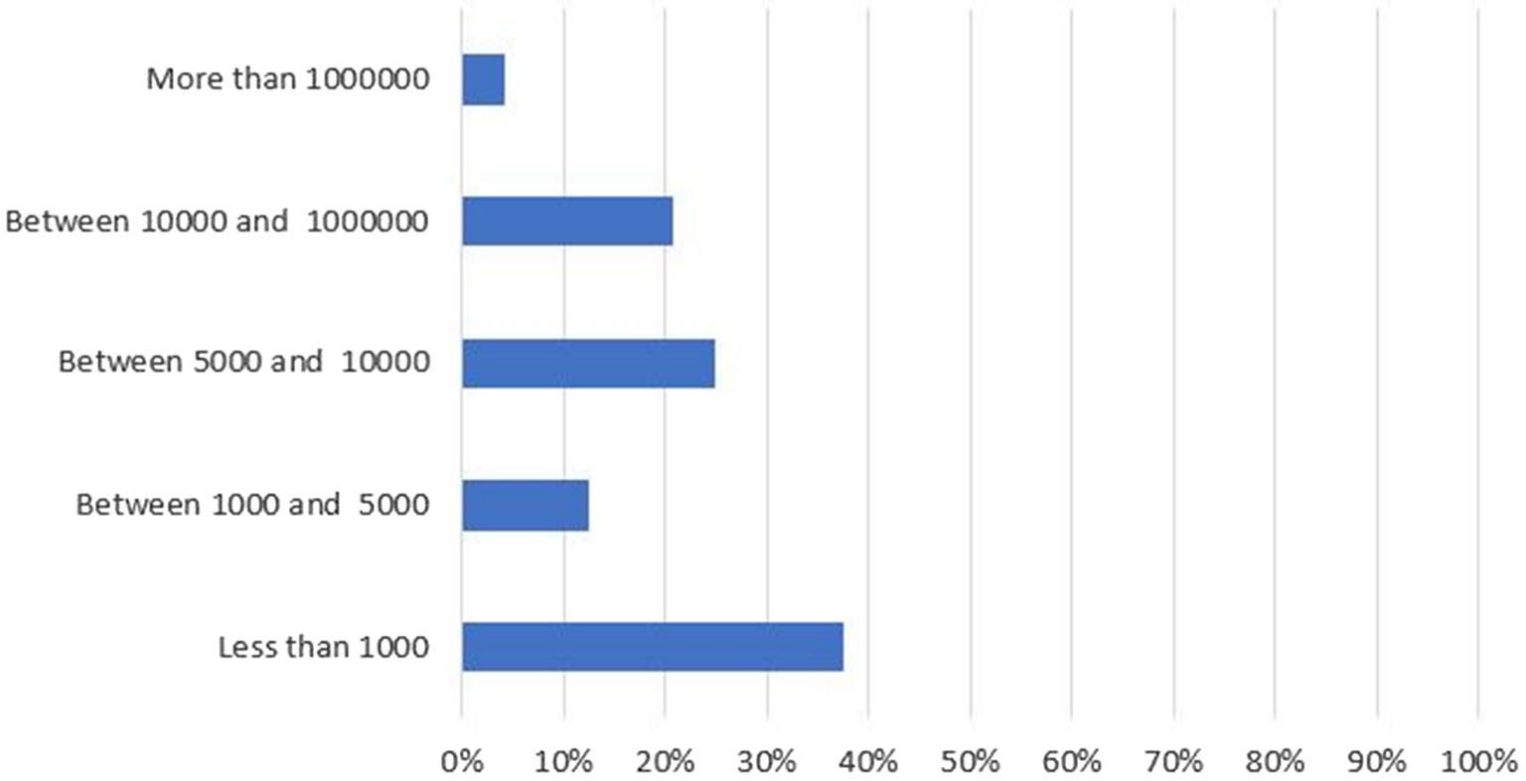
Percentage of downloads of the found apps.

As Fig. 4 shows, almost 40% of the apps found have less than a thousand downloads, and 50% have more than 5 thousand downloads. This means that Covid-19 related apps are being widely used among mobile device users at present.

### Summary of relevant app analysis

In the analysis performed, summarized in Table 2, the apps were classified according to their purpose in the categories of Information, Location/tracking, Self-assessment, Communication with physicians, and Outbreak tracker in the healthcare environment. In addition, a scale was created taking into account the following evaluation criteria to obtain the final ranking:

- Language (L): 2 points were awarded if the application was available in Spanish or English, 3 if both, and 4 if it had additional languages.
- OS: 2 points were assigned if the app was available only on Android or iOS; 4 if the app was available on both.
- Keywords (Kw): 1 was awarded for each keyword that resulted in the same app (range 1 to 4).
- Relevance (R): 5 points were distributed for each application according to user ratings.

**Table 2:** Categories and scores obtained by each app in the analysis.

Aplication	Category	Score				
		L	OS	Kw	R	Rk
COVID-19.eus	Information and case location/monitoring	3	4	3	3	13
GVA Responde	Self-evaluation	3	4	3	4	14
Asistencia COVID-19	Information, self-evaluation and health outbreak tracker	2	4	3	3	12
CoronaMadrid	Information, self-evaluation	2	4	3	3	12
CoronaTestNavarra	Information, self-evaluation	3	4	2	4	13
GVA Coronavirus	Information, self-evaluation	3	4	4	3	14
STOP COVID19 CAT	Information, self-evaluation	4	4	3	3	14
CONFINAPP	Information	4	4	4	4	16
OpenWHO: Knowledge for health emergencies	Information	3	4	3	3	13
WHO Info	Information	3	4	3	4	14
Radar COVID	Information, case location/monitoring and health outbreak tracker	3	4	3	4	14
Covclear	Self-evaluation, Case location/monitoring and health outbreak tracker	3	4	3	–	10
PassCOVID.gal	Information and self-evaluation	4	4	4	3	15
Ser + contra COVID19	Information	2	2	2	4	10
Salud Andalucia	Information	2	2	2	2	8
AyudaCovid	Information	2	2	3	2	9
HEpiTracker	Information and communication with doctors	2	2	2	2	8
Interactive Clinics	Information and communication with doctors	2	2	3	4	11
Mediknor	Information and communication with doctors	2	2	3	3	10
COVID-19!	Information	3	2	4	2	11
Coronavirus-Covid-19	Information	2	2	2	2	8
TousAntiCovid	Information and self-evaluation	2	2	4	2	10
Coronavirus-Extrem	Information	2	4	3	3	12
Salud Covid	Information, self-evaluation and communication with doctors	3	2	2	2	9

These evaluation criteria allow us to know the apps with the highest level of accessibility in terms of the number of languages supported, the operating systems and the number of appearances in keyword searches. The relevance criterion shows us the level of consumer acceptance of each of them and allows us to know the most accepted in the population.

To calculate the ranking (Rk) of the apps, the following formula was used (1):

1 $$Rk=L+OS+Kw+R$$

The apps with the highest scores were taken as a reference for the development of our proposal. It was detected that the apps that scored between 14 and 16 belong to the Xunta of Galicia, Generalitad Valenciana, Generalitad of Catalunya, WHO and the Ministry of Economic Affairs and digital transformation, which demonstrates the concern of governmental organizations in Spain in seeking solutions in periods of pandemic. The analysis found that there is no app that allows constant doctor-patient interaction without the need for real-time supervision by the physician.

### COVINFO app

The developed application is called COVINFO and its name comes from the union of the words COVID-19 and information, inserted in such a way that the user can get an idea of the functionality of the application by simply listening to or reading the resulting name. Version 1.0 of this app, as its source code, can be obtained from the GitHub repository^59^ under the free software license “GNU General Public License v3.0” which allows to commercial use, modification, distribution, patent use, and private use the code. In this way the users of this app can incorporate improvements depending on their specific needs, download the different versions of the app, access and rebuild the code for free.

The main function of the application is the transmission of the patients' symptoms to their respective doctors, thus avoiding the need for the doctor to call periodically to inquire about the presence of changes. In this way, the time needed by the doctor for each patient is reduced and makes it possible to attend to a greater number of patients in the same space of time.

The app has been created for mobile devices with Android operating system, being compatible from Android 4.4 onwards.

**Figure 5:**
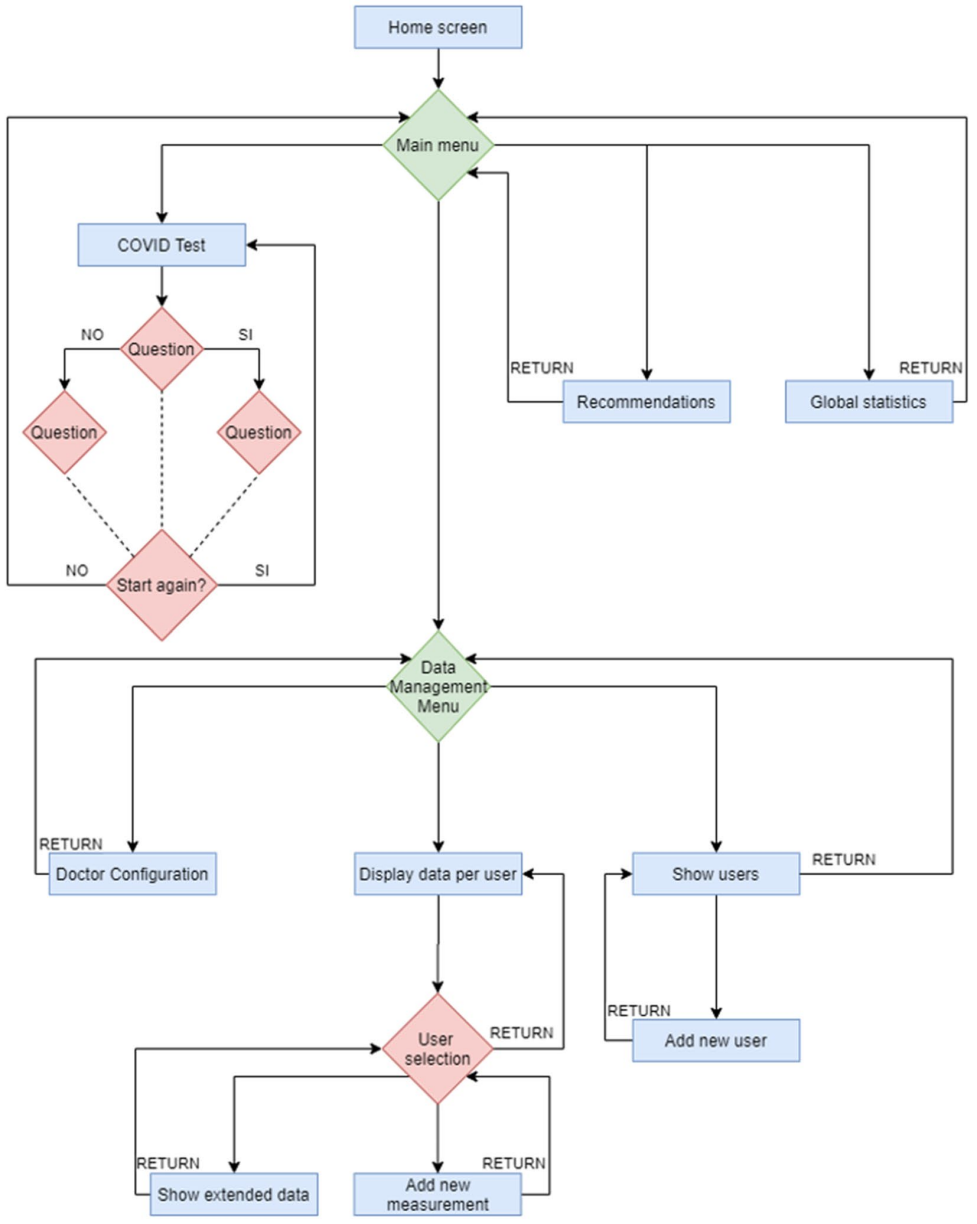
Flowchart of the different screens of the app.

Figure 5 shows the flowchart of the different screens of the application. It can be seen that the app has a welcome screen that goes to a selection menu with the options of Data Management, Covid Test, World Statistics, and Recommendations. Each of these options redirects the user to a new screen, each of which is explained below:

- COVID Test: The user can independently check if the symptoms he/she has are compatible with the virus.
- Worldwide statistics: Loads data updated daily by Google on COV-SARS-2 data from all over the world.
- Recommendations: The WHO recommendations to the population are shown.
- Data Management: All tasks related to sending symptomatology to the health center are performed. It also performs tasks related to the visualization of the data entered and their statistics.

When accessing the Data Management screen there are three distinct parts: (1) Display data per user which is used to visualize the information entered by the users, to add new measurements of the different users and to enable the data to be sent to the physician. (2) Display of the temperature graphs of the different users over time. (3) Modification of the assigned physician's data.

Another of the functionalities most requested by users, according to the functionalities of the most downloaded apps and the comments in the reviews of the apps found, is to know whether their symptoms are compatible with COVID-19. The Ministry of Health has enabled a phone number to contact and report if the symptoms are compatible with the virus, but this function has been automated so that the user only calls if the symptoms are really compatible with the virus. This self-assessment test is designed to help the user make the best decision in case of symptoms or doubts about SARS-COV-2. No data is collected during the test, and the result is strictly personal and confidential. At the end of the questionnaire, the user is informed about the compatibility or not of his/her symptoms with COVID, and the action to take in case of being compatible, to confine him/her at home and contact/communicate with a physician or in cases considered severe, e.g. respiratory distress, to call urgently the emergency department to receive recommendations as soon as possible.

In the Global Statistics screen, you get daily updated data uploaded by Google. Data are shown for all countries and, in addition, in the case of Spain, they can be segregated by autonomous communities. The data shown are the total number of cases in the selected area, the number of deaths and the number of vaccines administered. These data can be filtered by the last 14 days or since the beginning of the pandemic.

In Recommendations the most recent WHO recommendations for dealing with the pandemic are presented, and access to a hoax section has been added. This functionality is implemented to support the debunking of rumors that have been generated and that tend to generate unnecessary fears among users.

## Discussion

This section discusses the functionalities of the apps analyzed and compares the proposed app with those that have the same purpose, the limitations of the proposed application and the conclusions obtained in this study.

### Functionality and app comparison

**Figure 6:**
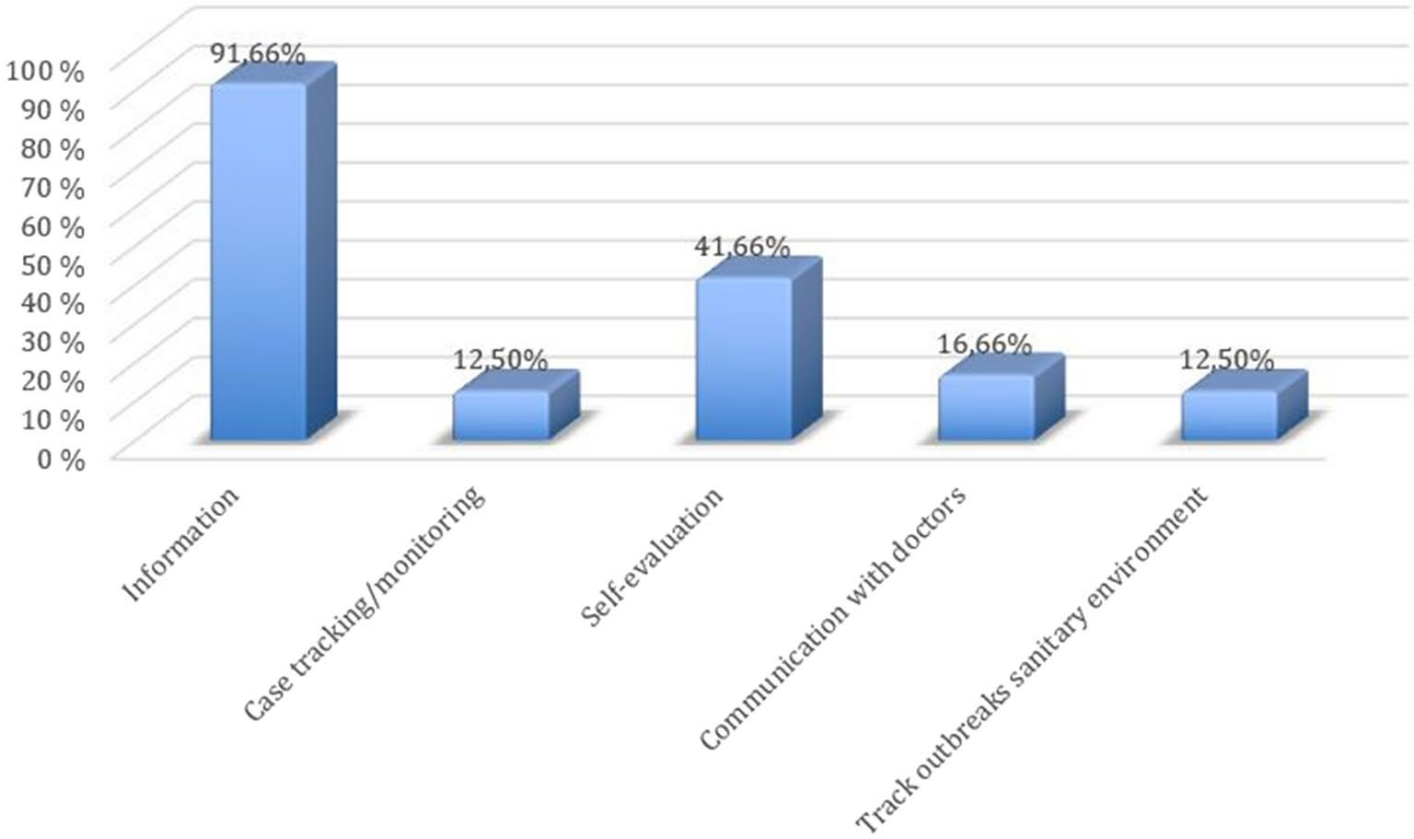
Percentage of apps by category.

Figure 6 show that 91.66% of the applications are or have a part dedicated to providing information and recommendations, generally linked to avoiding the spread of the virus (use of masks, hygiene, social distance, etc.). The 41.66% of the applications analyzed provide a self-assessment in which the user answers a series of questions about his/her symptoms and the system returns an estimated result of a possible COVID-19 based on the data entered by the user. This type of application does not exclude the user at any time from the PCR test in the event of probability or suspicion. Almost a fifth of the applications (16.66%) are intended to establish bidirectional communication with real physicians, who provide information and help at any given moment, avoiding the need to travel to the user's home. Only 12.5% of the applications analyzed have a use for locating or tracking contacts. On the other hand, according to some studies, in order for these applications to become useful, a minimum of 20% of mobile users need to implement them. But actually, after a few months of their implementation in Spain, it has been possible to glimpse that this type of applications has lacked utility and this is due to the low acceptance among the population to have them installed on the mobile. One of the most common reasons why most people do not install them is due to the fear of losing privacy.

Doctor-patient communication applications are designed as a real-time chat. Overcoming the limitations of this design is the main reason why in this work we have chosen to develop the COVINFO application, which allows asynchronous doctor-patient communication, while patients send their measurements so that the doctor can review them without waiting. The COVINFO app sends an e-mail summary of the patient's follow-up to the corresponding physician once the patient updates his daily symptomatology. COVINFO presents the option of setting reminder alarms to the patient to obtain a periodic follow-up of the symptomatology. This option allows establishing short or long follow-up periods as the physician deems appropriate, depending on the patient's evolution.

Table 3 shows a comparison of apps with the same purpose as COVINFO, communication with physicians. It can be seen that our app presents novel functionalities, since it allows asynchronous communication, graphs and history of the patient's evolution and reminders at specific time periods for effective follow-up.

**Table 3:** Apps comparison.

App	Information	Self-evaluation	Communication asynchronous	Follow-up of the patient's evolution	Time periods determined for effective monitoring
HEpiTracker	✓	−	−	−	−
Interactive Clinics	✓	−	−	−	−
Mediknor	✓	−	−	−	−
Salud Covid	✓	✓	−	−	−
COVINFO	✓	✓	✓	✓	✓

### Limitations of the COVINFO app

The COVINFO app is an app that is still in the testing and development phase. It has limitations such as language, only in Spanish; the OS, it is only for devices with Android OS; the access, because it is in the testing phase, currently the app is not in app stores, nor in free access repositories; information storage, despite being one of its strength storage in an internal database, so it does not require internet connection for its use, it also represents a limitation in case of loss or theft of the mobile device; data security also represents a limitation in the process of being resolved in this app, since in case of theft of the device the user's data would be in clear and unprotected, we are working to implement an access screen to the app that requires biometric data of the user.

## Conclusions

In this research, a search for apps related to the Covid-19 pandemic available for Spain has been carried out, resulting in a total of 24 apps, either iOS or Android OS. In addition, the development of an application for the transmission of the user's symptoms to their regular doctor is addressed, since only 16.6% of existing applications have this functionality. The COVINFO app offers a service that no other application on the market has: doctor-patient interaction without the need for calls or chat in real time for constant monitoring by the doctor of the patient's condition and evolution. Another of the purposes of this application is for users to enter their measurements even if they do not have symptoms, so that if at any time they start to have symptoms, a history of how the virus affects the different users can be made and, in this way, useful experiences can be taken for future patients.

To provide a more complete service with the COVINFO app, we have set as future work to implement the communication of the internal database (present in the mobile devices where the COVINFO app is installed), with an external database, to avoid the loss of information due to the loss of the mobile device; implement an access screen to the app that requires biometric data from the user to guarantee the security of the information, as well as encryption algorithms to guarantee the storage and transfer of the encrypted information; implement the app in at least two languages and for iOS OS; add new histograms of the data entered by the patient and incorporate them into the physician's email for better visualization of the data; add a new option on the data submission screen that allows the user to choose the range of dates they wish to send to the health center; perform multiple tests in a real environment to provide support and updates to the app before releasing it to the market; after testing and validation of the app by patients and healthcare personnel. In addition, in the following versions of the app we intend to integrate the information obtained with Electronic Medical Record (ERM) systems and provide multichannel communication (text messages, emails, etc.) according to the needs of each hospital system.

## Data Availability

The data analyzed in this study were obtained from the Google Play Store and App Store mobile app stores. The code of the created app is not available in public repositories.
